# Shape Optimization of Rubber Bushing Using Differential Evolution Algorithm

**DOI:** 10.1155/2014/379196

**Published:** 2014-09-03

**Authors:** Necmettin Kaya

**Affiliations:** Mechanical Engineering Department, Engineering Faculty, Uludag University, 16080 Bursa, Turkey

## Abstract

The objective of this study is to design rubber bushing at desired level of stiffness characteristics in order to achieve the ride quality of the vehicle. A differential evolution algorithm based approach is developed to optimize the rubber bushing through integrating a finite element code running in batch mode to compute the objective function values for each generation. Two case studies were given to illustrate the application of proposed approach. Optimum shape parameters of 2D bushing model were determined by shape optimization using differential evolution algorithm.

## 1. Introduction

Bushings are used in automotive industry for vibration isolation and comfort requirements. They are produced from rubber materials and their main functions are to join the elements between rigid structures, isolate vibrations through to the chassis, and avoid the transmission of noise in the vehicles. Due to the increasing interest of multibody simulations of complete vehicles or subsystems, it is important to develop and effective models to represent the static stiffness of these rubber products in vehicles. During the vehicle development process, shape optimization of rubber products is also need to have target stiffness curves. Many bushing manufacturers use trial and error method to meet these requirements, but optimization algorithms are the solution to this type of design problems. This paper presents a simulation-based approach to optimize two-dimensional rubber bushing model to meet target radial static stiffness without the need for physical prototypes.

Rubber bushings are used mostly in vehicle suspensions. The primary role of the bushings in a suspension system is to improve the ride quality of the vehicle. Their stiffness curve has been primarily research subjects for many researchers. Blundell [1] concluded that suspension designs depend on the behavior of rubber bushings. He described the influence of rubber bushing compliance on changes in suspension geometry during vertical movement relative to the vehicle body. An experimental investigation was conducted on elastomeric bushings, which was presented by Kadlowec et al. [2, 3]. The experiment reveals that the relationship between the forces and moments and their corresponding displacements and rotations is nonlinear and viscoelastic due to the nature of the elastomeric material. A parameter identification method by Lei et al. [4] is proved to model the appropriate hyperelastic material for rubber bushing validly when material tensile tests data are not provided. In terms of the hyperelastic material of this bushing, three-term Ogden law is utilized as the material constitutive model.

Recently, the use of non-deterministic algorithms has attracted the researchers to find global optimum. Among the nondeterministic methods, the differential evolution (DE) algorithm produced good results in the literature for different applications in science and engineering. DE and particle swarm optimization methods have been applied to the design of minimum weight toroidal shells subject to internal pressure. The optimization process is performed by Fortran routines coupled with finite element analysis code Abaqus [5]. An investigation into structural topology optimization using a modified binary DE with a newly proposed binary mutation operator is performed [6]. Carrigan et al. [7] introduced and demonstrated a fully automated process for optimizing the airfoil cross-section of a vertical-axis wind turbine using a parallel DE algorithm. Jena et al. [8] presented a damage detection technique combining analytical and experimental investigations on a cantilever aluminium alloy beam with a transverse surface crack. The damage location is formulated as a constrained optimization problem and solved using the DE algorithm based on the measured and calculated first three natural frequencies as inputs. A framework for the shape optimization of aerodynamics profiles using computational fluid dynamics and genetic algorithms proposed by López et al. [9]. A DE optimization based technique is proposed to find the optimum value of a modified Bezier curve. The proposed equation contains shaping parameters to adjust the shape of the fitted curve [10]. Ketabi and Navardi [11] proposed a new method for optimum shape design of variable capacitance micromotor using DE algorithm. The objective function aims to maximize torque value and minimize the torque ripple, where the geometric parameters are considered to be the variables. The optimization process is carried out using a combination of DE algorithm and FEM analysis.

Shape optimization of rubber mounts and bushings was performed by many researchers. A parameter optimization methodology for a rubber mount based on finite element analysis (FEA) and genetic neural network models is proposed in [12]. Through a combination of FEA and genetic neural network methods, the parameters of the rubber mount were optimized to meet the design requirements. The optimum nonlinear stiffness curve of rubber suspension was obtained through the whole vehicle dynamics optimization using classical optimization algorithms such as sensitivity analysis and sequential quadratic programming theory in [13]. Ambrósio and Verissimo [14] discussed the sensitivity of the ride characteristics of a road vehicle to the mechanical characteristics of the bushings used in its suspension. Sensitivities of different vehicle kinematic responses to the characteristics of the bushings used in the suspension are evaluated, by using numerical sensitivities. A bush type engine mount has been designed using a parameter optimization method in [15]. An optimization code is developed to determine the shape to meet the stiffness requirements of engine mount, coupled with commercial nonlinear finite element program. Powell's penalty function method was used as optimization algorithm. Lee and Youn [16] proposed a topology optimization for the design of rubber vibration isolators. The topology optimization formulation is proposed in order to generate the system layouts considering both the static and dynamic performance. The density distribution approach and sequentially linear programming were used as the optimization algorithms.

Less attention has been paid to optimization of the rubber products to have target nonlinear stiffness curve in the literature. Main contributions of this paper are as follows.

- The parameters of rubber bushing were optimized to obtain desired level of stiffness.
- A DE based global optimization software was developed and tested with two test functions.
- Rubber material experiments were performed to obtain hyperelastic model coefficients.

In this study, a methodology for determination of shape parameters of a rubber bushing to have a desired stiffness curve has been proposed. Shape optimization was used to design a two-dimensional rubber bushing model using DE algorithm to meet target stiffness curve. Stiffness curves of rubber bushing with different geometric parameters in radial directions were obtained by finite element method. A Pascal (in Delphi environment) code based DE was developed for shape optimization. Developed optimization software was tested with two test functions; then, optimization process was performed using a combination of DE algorithm and FE analysis.

## 2. Differential Evolution Algorithm

One of the main shortcomings of classical optimization methods is to stuck into local optimum instead of global optimum. Genetic algorithm and differential evolution algorithms are evolutionary optimization algorithms; they were developed for finding the global optimum of the optimization problems. DE is a relatively new evolutionary optimization algorithm. It is a population-based optimization method introduced by Price et al. [17]. They developed a new robust, versatile, and easy-to-use global optimization algorithm and published it under the name differential evolution (DE) algorithm in 1995. This algorithm, like other evolutionary algorithms, has a population-based structure, and it attacks the starting point problem using a real-coded system and a new differential mutation operator. The DE algorithm's main strategy is to generate new individuals by calculating vector differences between other individuals of the population. The DE algorithm includes three important operators: mutation, crossover, and selection. In the DE, population vectors are randomly created at the start of iteration. This population is successfully improved by applying mutation, crossover, and selection operators, respectively. Mutation and crossover are used to generate new vectors (trial vectors), and selection then is used to determine whether or not the new generated vectors can survive the next iteration. Among the strategies in DE algorithm, DE/rand/1/bin DE strategy was used. The details of DE algorithm are given below.

DE was firstly proposed for minimizing unconstraint real single objective optimization. DE consists of two fundamental phases: initialization and evolution [18]. In the initialization phase, just like in other evolutionary algorithms, an initial population (*P* ^0^) is generated. After that, the *P* ^0^ population evolves to *P* ^1^, *P* ^1^ evolves to *P* ^2^, and so on. In this way, evolution of new populations is continued until the termination conditions are fulfilled. While evolving from the *P* ^*n*^ to *P* ^*n*+1^, three evolutionary operations are executed on the individuals in the current population. These operations are differential mutation, crossover, and selection [18].

### 2.1. Initialisation

In this stage, the initial population *P* ^0^ is randomly created from *N*_*p*_ number of individuals:

(1) $$\begin{array}{c} x_{j}^{0,i}=b_{j}^{L}+\alpha_{j}^{i}\left(b_{j}^{U}-b_{j}^{L}\right),\;1\leq j\leq N_{p}, \end{array}$$

where 0 means the initial population, *i* is the sequence of the population, *j* is the number of individuals in the population, *α*_*j*_^*i*^ is the real random number generator in the *i*th population and *j*th individual, *b*_*j*_^*L*^ is the lower value of the *j*th individual, and *b*_*j*_^*U*^ is the upper value of the *j*th individual.

### 2.2. Differential Mutation

In mutation, a mutant (*v* ^*n*+1,*i*^) and a mutant vector (*x* ^*n*+1,*v*,*i*^) are created for each *p* ^*n*,*i*^ individual, called a mother, in the *P* ^*n*^ population. It should not be forgotten that *x* is a vector that represents all individuals in the current population (*x* = *x*_1_, *x*_2_, … , *x*_*N*_).

Mutant vector *x* ^*n*+1,*v*,*i*^ is created as follows:

(2) xn+1,v,i=xn,b,i+∑y≥1Fy(xn,p1y−xn,p2y),1≤i≠p1y≠p2y≤NP,

where *x* ^*n*,*b*,*i*^ is the base vector (*b*) selected for the new individual that will be created for the *i*th old individual in the *n*th population and *x* ^*n*,*P*1,*i*^ is the *P*_1*y*_th individual selected randomly from between [1, *N*_*P*_] integers. Similarly, *x* ^*n*,*P*2,*i*^ is the *P*_2*y*_th individual selected randomly from between [1, *N*_*P*_] integers, and *F*_*y*_ is the scale factor for the *y*th vector difference in the range of [0,1].

The *x* ^*n*,*b*,*i*^ base vector can be selected in different ways:

- from the current vector: *x* ^*n*,*b*,*i*^ = *x* ^*n*,*i*,*i*^, (*b* = *i*);
- from the best vector: *x* ^*n*,*b*,*i*^ = *x* ^*n*,best,*i*^, (*b* = the best);
- from the better vector: *x* ^*n*,*b*,*i*^ = *x* ^*n*,better,*i*^, (*b* = the better);
- from a random vector: *x* ^*n*,*b*,*i*^ = *x* ^*n*,random,*i*^, (*b* = random).

After the mutation process, the new individual can be created outside the range of [*b*_*j*_^*U*^, *b*_*j*_^*L*^]. Various methods have been proposed for infeasible individuals [18].

### 2.3. Crossover

In this process, a new child individual (*c* ^*n*+1,*i*^) is created by mating the new individual (*x* ^*n*+1,*i*^) that is created in the mutation process with the current individual (*p* ^*n*,*i*^) in the population according to the crossover probability *C*_*r*_. Here, *p* ^*n*,*i*^ is referred to as the mother, and *x* ^*n*+1,*i*^ is referred to as the father.

### 2.4. Selection

There is a competition between mother and child in the selection operation. They compete with each other according to objective function values to survive in the next generation [18]. This competition is formulated mathematically as follows:

(3) $$\begin{array}{c} p^{n+1,i}=\{\begin{array}{cc} c^{n+1,i}, & \text{if}\;\;\left(c^{n+1,i}>p^{n,i}\right),\\ p^{n,i}, & \text{otherwise}. \end{array} \end{array}$$

The key parameters of control in DE are as follows:

 
*N*_*P*_: the population size (number of individual);
 
*C*_*r*_: the crossover constant (probability) (0.0-1.0);
 
*F*_*y*_: scaling factor that controls the amplification of differential variations (0.0–2.0).

During the iterations of DE algorithm, various feasible and unfeasible individuals may appear. Regular DE operators can produce unfeasible individuals. It means that some individuals may violate the constraints. For example, at some stage of the evolution process, a population may contain some feasible and unfeasible individuals. Therefore, several trends for handling unfeasible solutions have emerged in the area of evolutionary computation. In this study, Schoenauer and Xanthakis's method was adopted [19]. In this method, any individual do not complying with the constraints is eliminated and a new individual is created. This insures that the size of the population remains constant even when eliminating those individuals violate the constraints. Therefore, every individual in the population satisfies the constraints.

In this study, DE algorithm was selected for shape optimization due to following reasons [20].

- It finds the lowest fitness value for most of the problems.
- DE is robust; it is able to reproduce the same result consistently over many trials.
- It is simple and robust, converges fast, and finds the optimum in almost every run.

DE algorithm is slower than the other evolutionary algorithms especially for noisy problems. This is the disadvantage of the DE algorithm.

Pascal programming language based DE optimization software was developed and validated using two test functions. After validation of the developed DE optimization software, optimum shape parameters of 2D rubber model were determined using differential evolution optimization algorithm.

## 3. Optimization of Test Functions with DE

The developed DE software was validated using two test functions. The first test function is an unconstrained function called Rosenbrock's saddle [21]. It is a nonconvex function used as a performance test problem for optimization algorithms introduced by Howard H. Rosenbrock in 1960. It is also known as Rosenbrock's valley or Rosenbrock's banana function. The global minimum is inside a long, narrow, parabolic shaped flat valley. However, it is difficult to find the global optimum of this function with the classical optimization algorithms.

The function and bound values of parameters are given as

(4) min⁡F(x1,x2)=100(x12−x2)2+(1−x1)2 − 2.048≤x1, x2≤2.048.

**Figure 1:**
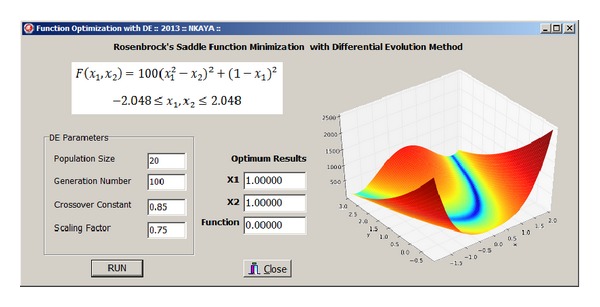
Optimization user interface and results for test function 1.

It has a global minimum at (*x*_1_, *x*_2_) = (1, 1) where *f*(*x*_1_, *x*_2_) = 0 [19]. Population size of 20, generation number of 100, and the crossover constant and scaling factor of 0.85 and 0.75 were selected. User interface of the software for test function is given in Figure 1.

Results were obtained as *x*_1_ = 1 and *x*_2_ = 1 exactly. It can be seen that the DE successfully converged to the global minimum easily. It is clear from the generation history that generation number as 40 is enough, because it converged at the 40th iteration.

Having validated the first unconstrained test function, DE software was also validated using second test function which is constrained type and it is taken from [22]. The objective function and constraints are given as follows:

(5) min⁡ F(x1,x2)=(x1−10)3+(x2−20)3s.t.  g1(x1,x2)=−(x1−5)2−(x2−5)2 +100≤0   g2(x1,x2)=(x1−6)2+(x2−5)2−82.81≤0,

where 13 ≤ *x*_1_ ≤ 100 and 0 ≤ *x*_2_ ≤ 100.

This optimization problem has a global minimum at (*x*_1_, *x*_2_) = (14.095, 0.84296) where *f*(*x*_1_, *x*_2_) = −6961.8 [22]. The DE software is also validated with the second test function, because the same results were obtained as in the literature. It is concluded that developed DE software can be used for subsequent shape optimization studies.

## 4. Finite Element Modeling of Rubber Bushing

Rubber bushings in a vehicle suspension system can affect the stability of the vehicle. Thus, the stiffness characteristic of a rubber bushing in each direction is achieved by analyzing the vehicle performance during the design process. To design a particular rubber bushing, the stiffness in certain direction needs to meet the requirements. The purpose of this paper is to make the radial stiffness characteristic of the given 2D rubber bushing model meet the target stiffness curve by using the optimization method presented in this paper. In order to determine the stiffness curve of the bushing, nonlinear finite model was defined using Abaqus software [23].

### 4.1. CAD-Based Design Parameterization

The parametric 2D CAD model is used for design optimization. Dimensions are geometric parameters that can be varied permitting design change while preserving the basic shape or design intent of the part. Automatic model regeneration is an essential feature of dimension-driven systems. If a dimension is changed, the model should be regenerated automatically while preserving geometric constraints and relationships. Design intent will be captured by establishing and preserving these relationships [24].

**Figure 2:**
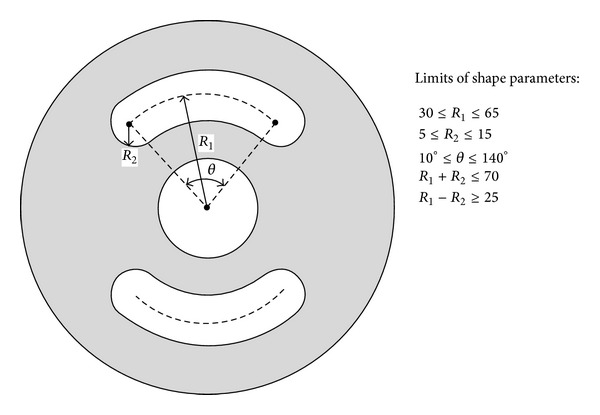
2D rubber parametric model and limits of shape parameters.

Three parameters were selected as design variables for 2D bushing model as shown in Figure 2. The guiding principle to select these design variables is to choose those parameters which influence the rubber bushing stiffness characteristics most. Also limits and constraints were defined in order to preserve the shape. In order to undergo further parametric optimization, Python programming language was used to build the model in Abaqus software in terms of parameters such as *R*_1_, *R*_2_, and *θ* as shown in Figure 2.

### 4.2. Material Model

The natural rubber can be considered as a hyperelastic material, showing highly nonlinear elastic isotropic behavior with incompressibility. A relationship between stress and strain in the hyperelastic material, generally characterized by strain energy potentials, is essential for the FEA of rubber components. In order to define the hyperelastic material behavior, that is, the constitutive relation, experimental test data are required to determine material parameters in the strain energy potential. In this study, uniaxial and planar tension tests were performed at a local rubber company.

**Figure 3:**
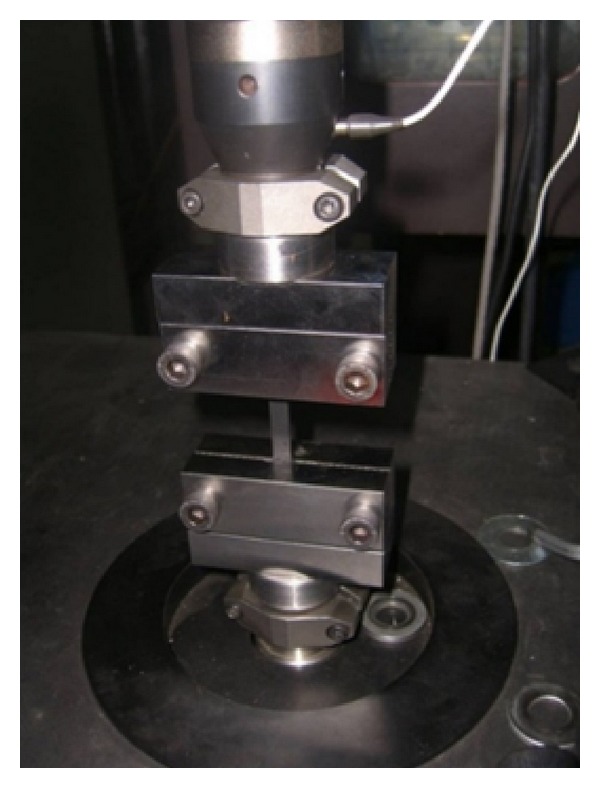
Uniaxial tension material test.

Uniaxial test is the main test to achieve pure tension effect. The extended length of sample must be sufficiently long in the direction of stretching compared with the width and thickness. For this reason, the length of the sample must be ten times greater than the width. The measurement is done from only the pure tension area that is the distance between two chins. The uniaxial test condition is given in Figure 3.

In uniaxial tension test, the material properties vary dramatically at first cycles. This behavior is called “Mullin effect.” After the number of certain cycles (from 3–20), the material shows a stable behavior. If the material is exposed to a different high tension, after a few cycles, it will show a stable behavior again. Mullin effect is considered for the tests done in this research.

**Figure 4:**
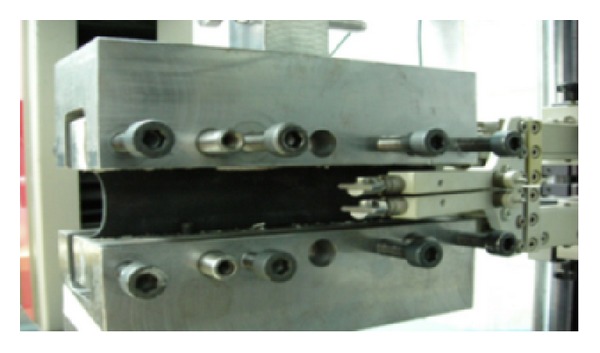
Planar tension material test.

Planar tension test resembles the uniaxial tension test but the sample length is smaller than the width (Figure 4). Thinning of the sample is along the thickness direction. Because of the fact that rubber is incompressible, pure shear arises at 45° angle of tension direction. To provide this event, the width of the sample must be ten times greater than the length. The specific holders are used to overcome the specimen slippage from the clamp edges since this may lead to the inadequate states of pure shear strain. Therefore, a special gripping device, which is shown in Figure 4, is designed to prevent specimen slippage in order to improve the test accuracy.

These two test data are evaluated in Abaqus software and compared to the hyperelastic material models.

**Figure 5:**
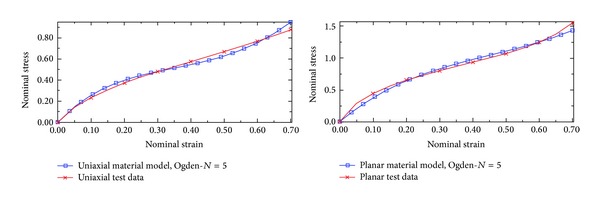
Rubber material for test data (stress-strain curve).

Among the hyperelastic materials models, the Ogden *N* = 5 produces a better fit for test data as shown in Figure 5. The Ogden material model appears to best capture the bushing response in the finite element study. Therefore, this material model was selected in the finite element model.

### 4.3. FE Analysis of 2D Rubber Model

The proper element type and reasonable meshing strategy were used to model the two-dimensional rubber model. The central and lower parts of the rubber component will come in contact when the slot is closed under vertical displacement applied to the center of the rubber. Therefore, contact interaction was defined in the finite element model. Thus, this becomes a nonlinear large displacement contact analysis. As a whole, the initial finite element model has about 1200 hybrid 2D elements (CPE4H) and 1340 nodes.

**Figure 6:**
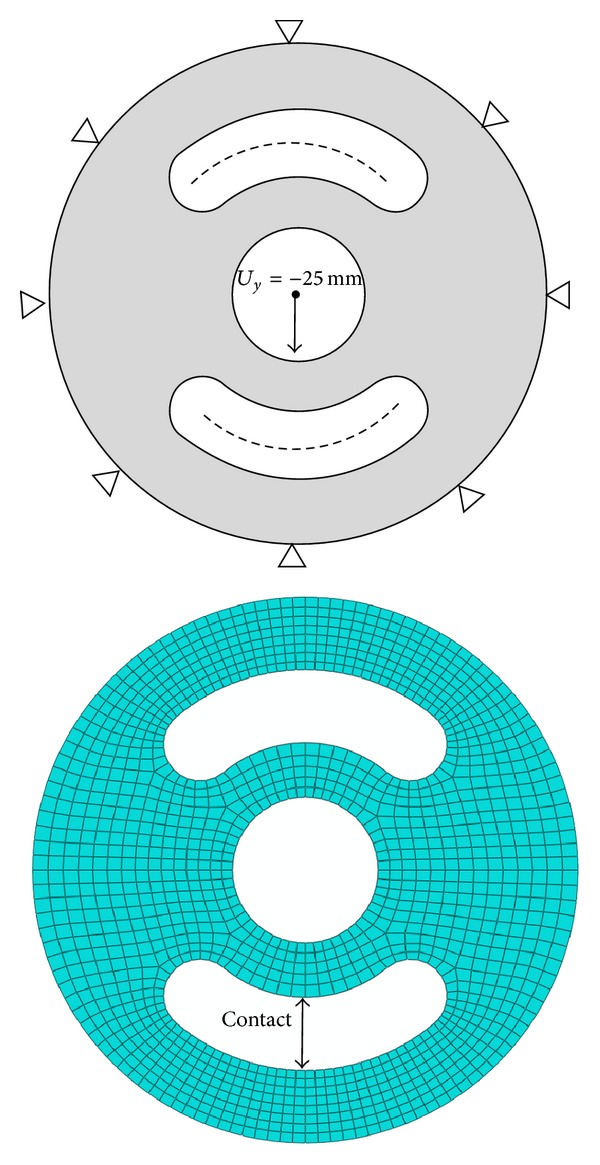
Boundary conditions and finite element model of bushing.

As shown in Figure 6, the displacement of 25 mm was applied from the center of the inner circle using rigid connection and self-contact was defined for lower slot. Outer circle is constrained with all degrees of freedom. Reaction forces were stored in a text file for every displacement iteration during solution process.

**Figure 7:**
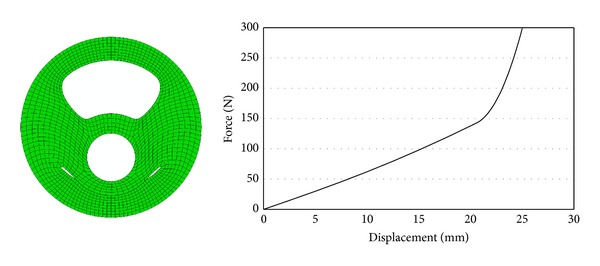
Deformed model and radial stiffness curve.

Deformed model and radial stiffness curve obtained after the solution are given in Figure 7.

During radial deformation, the slope of stiffness curve slowly increased; after closing the lower slot by self-contacting, it suddenly increased.

## 5. Differential Evolution Based Shape Optimization

The objective of the shape optimization of rubber bushing is to find the shape parameters that provide the desired static stiffness curve.

In this study, the difference between the calculated data point (*F*_calc_) and the target (*F*_target_) data point for each point in a stiffness curve is measured by a statistical term called Chi-square which is given as follows:

(6) $$\begin{array}{c} \text{chi-square}=\overset{n}{\underset{i=1}{\sum }}\frac{{\left(F_{\text{calc}\_i}-F_{\text{target}\_i}\right)}^{2}}{F_{\text{target}\_i}}. \end{array}$$

**Figure 8:**
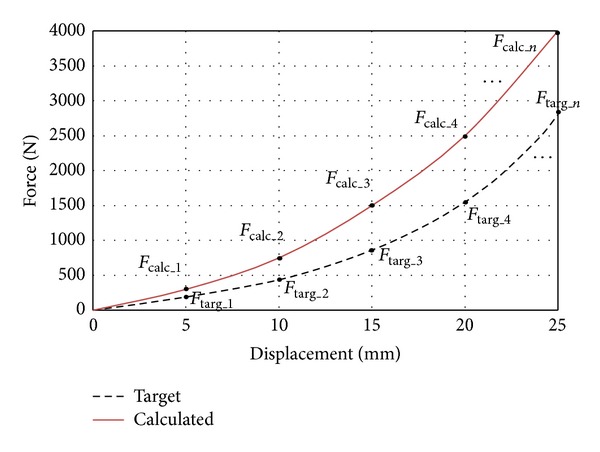
Chi-square calculation points.

Here, *n* is the measurement points shown in Figure 8.

If the Chi-square is large, then the calculated and target curves are not close to each other. If the two curves are exactly the same, Chi-square will be zero. The large value means that two curves are not identical and very close from each other. In this study, Chi-square must be as small as possible to have the desired static stiffness of rubber bushing.

*F*_calc_ refers to the stiffness curve of any individual in DE algorithms and is calculated by finite element analysis.

In this study, minimization of Chi-square was selected as objective function. Therefore, the shape optimization problem was defined as follows:

Objective is

(7) min⁡ ⁡chi-squares.t.  30≤R1≤65,  5≤R2≤15,10°≤θ≤140°   R1+R2≤70,  R1−R2≥25.

Shape optimization of two-dimensional bushing was implemented using software written in Pascal programming language. Displacement and forces which are required to obtain the radial static stiffness curve are calculated in Abaqus finite element analysis software. Abaqus finite element model is able to perform batch jobs when giving a parameter vector, the complete simulation, including geometric modeling, meshing, and postprocessing. The simulation results are performed in a fully automatic manner using Python programming language. Developed software sends shape parameters to Abaqus to analysis and generates stiffness curve. After the solution is obtained, displacements and forces at the circle center are written to a text file. DE algorithm reads this file and computes the objective function (fitness).

**Figure 9:**
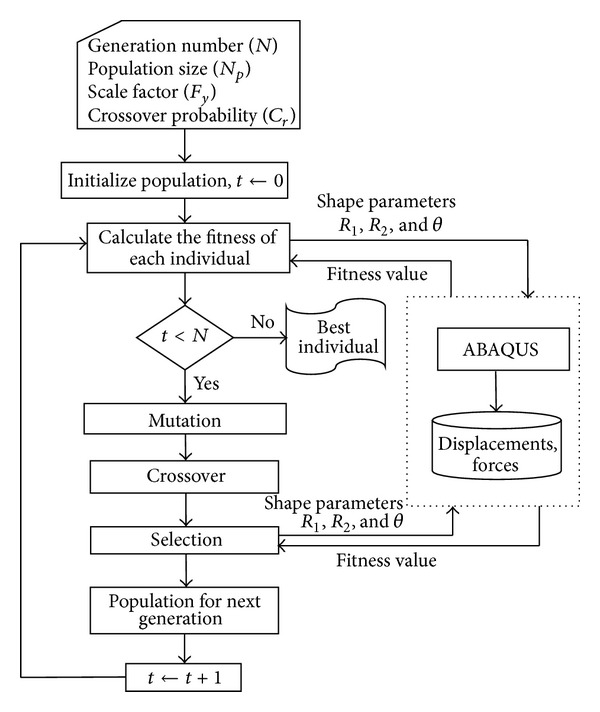
The flowchart of the proposed methodology and Abaqus interface.

Shape optimization of the rubber bushing using proposed methodology is shown in Figure 9. Two case studies were given below for shape optimization. In these case studies, target stiffness curves which are expected from rubber design were defined as a polynomial form. Chi-square value was calculated using actual and target stiffness curves for objective function.

**Figure 10:**
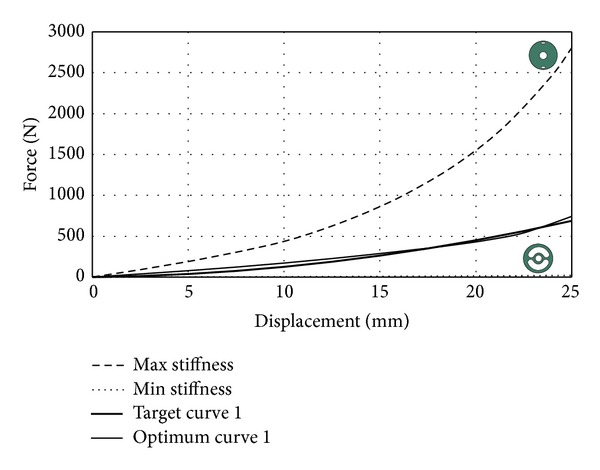
Optimization results for target curve 1.

*Case Study 1*. Before setting a target stiffness curve, it should be known of limits the stiffness curves as shown in Figure 10. Target stiffness curves should remain between maximum and minimum stiffness curves. For this case study, target stiffness curve can be seen in Figure 10.

**Figure 11:**
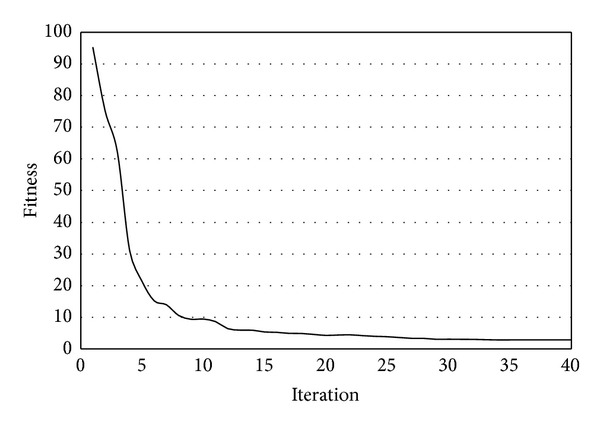
Iteration history for case study 1.

This optimization problem was solved by DE optimization algorithm. DE parameters were selected as follows: population size: 20; number of generations: 40; scale factor: 0.75; crossover rate: 0.85. After the solution phase completed, optimum shape parameters were found as *R*_1_ = 50, *R*_2_ = 12, and *θ* = 27. As seen in Figure 10, optimum curve is very close to target curve. Thus, DE algorithm was successfully applied to shape optimization problem in this case study. Iteration history for this case study is given in Figure 11.

**Figure 12:**
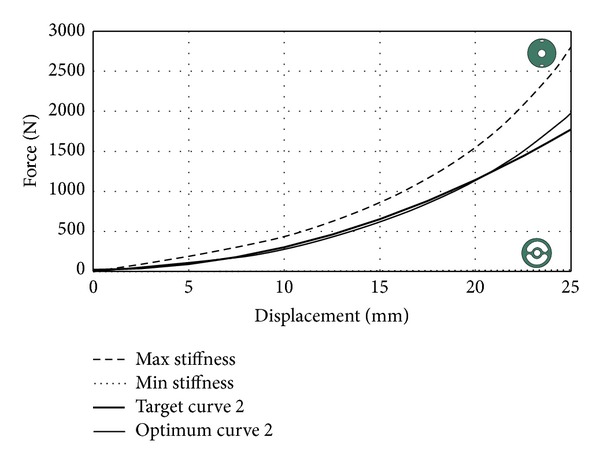
Optimization results for target curve 2.

*Case Study 2*. In the second case study, stiffer rubber bushing compared to case study 1 is expected. Target stiffness curve is shown in Figure 12.

DE parameters were selected same as for case study 1. After solving the optimization problem with DE algorithm, optimum shape parameters were found as *R*_1_ = 44, *R*_2_ = 5.5, and *θ* = 29. As seen in Figure 12, optimum curve is close to target curve 2 again. In this case study, optimum curve is the best one that matches the target curve.

Proposed methodology was successfully applied to shape optimization problem. It may be considered that this gives a systematic guidance to the shape designer of bushing. By a similar method, this approach can be used in the design of other types of rubber products such as engine mount and so forth in the automotive industry.

## 6. Conclusion

In this study, a differential evolution algorithm based shape optimization is presented. A Pascal code based on DE algorithm was developed to solve shape optimization problems. DE algorithm was successfully applied to shape optimization of 2D rubber bushing to obtain target stiffness curves. Abaqus software was used for the FE calculation of objective function. It is seen that the combined DE algorithm and FE method approach seem to be powerful for finding a global optimum. The DE method is particularly suited to problems where there is no well-defined mathematical relationship between the objective function and the design variables. The proposed method can shorten the rubber products design cycle and decrease the trial-and-error efforts.
